# Uptake of the Ithaka mobile application in Johannesburg, South Africa, for human immunodeficiency virus self-testing result reporting

**DOI:** 10.4102/sajhivmed.v22i1.1197

**Published:** 2021-02-22

**Authors:** Alex E. Fischer, Mothepane Phatsoane, Mohammed Majam, Luke Shankland, Musaed Abrahams, Naleni Rhagnath, Samanta T. Lalla-Edward

**Affiliations:** 1 Ezintsha, Faculty of Health Science, University of the Witwatersrand, Johannesburg, South Africa; 2 Aviro Health, Cape Town, South Africa

**Keywords:** HIV, HIV self-test, self-reporting, mobile app, mHealth, monitoring and evaluation

## Abstract

**Background:**

Human immunodeficiency virus self-testing (HIVST) can reduce facility-based HIV testing barriers; however, no proven applications exist with widespread uptake for self-reporting or linkage to care. Mobile health (mHealth) applications (apps) have shown high usability and feasibility scores, so Ithaka was developed for South Africans to self-report HIVST results outside clinical settings.

**Objectives:**

This study investigated the use of Ithaka as a support tool for HIVST users, specifically the ability to self-report results.

**Method:**

This cross-sectional study was conducted from November 2018 to June 2019. At existing HIVST distribution sites, individuals were given HIVST kits and then invited to use Ithaka. Participants could test at home and report their results through the app anytime. Ithaka tracked when people logged-on, registered, received counselling and reported results. Post-study surveys on user experience were also conducted.

**Results:**

Of 751 participants, 531 (70.7%) logged onto the app, 412 (54.9%) registered, 295 (39.3%) received counselling and 168 (22.4%) self-reported results. Participants strongly agreed that Ithaka was useful and that it was easy to upload results. Forty-one participants completed a post-test survey, and 39/41 (95.1%) completed the app journey. Most participants (36/41;87.8%) had no challenges, although 2/41 (4.9%) cited perceived data costs, 2/41 (4.9%) difficulty uploading results and 1/41 (2.4%) language, as challenges.

**Conclusion:**

Despite the small sample size, this study has shown that HIVST participants under pragmatic conditions were willing and able to self-report results via the app, whilst also identifying areas of improvement for scaling up.

## Background

Human immunodeficiency virus self-testing (HIVST) can reduce barriers associated with conventional facility-based HIV testing, and since its introduction in 2012, more than 6.5 million HIVST kits have been distributed globally.^1,2^ In 2018, South Africa integrated HIVST into its national HIV strategy as a way to expand testing beyond standard healthcare facilities to meet the UNAIDS 90-90-90 target.^3,4^ These targets state that 90%, 81% and 73% of the total population should know their HIV status, be linked to antiretroviral treatment (ART) and experience viral suppression, respectively.^5^ Despite the benefits of HIVST, there are some gaps associated with its use, as it is hard to track and is only classified as tests for triage, which should not be considered diagnostic.^6^

Furthermore, South Africa does not have an appropriate system for users to self-report their results, or be linked to care, and this lack of reporting makes it difficult for public health stakeholders to conduct monitoring and evaluation on the uptake and effectiveness of HIVST, especially at the population level.^7^

Over the last decade, low- and middle-income countries have experienced an increase in mobile coverage and smartphone use, which has qualified the introduction of mobile health (mHealth) interventions in these regions.^8,9,10^

There is a strong body of evidence supporting the use of mHealth interventions to enhance patient outcomes for a broad spectrum of health conditions, including HIV. In low-income settings specifically, different interventions have targeted various stages of the HIV care cascade, including text message campaigns, telephone hotlines and mobile applications (apps).^6,11,12,13,14^

South Africa has been investigating the use of mHealth interventions to accompany HIVST for users to self-report their results, and in a recent study, 9.8% of participants self-reported their results by using an interactive voice response telephone hotline.^15^ Feasibility studies have also been done on the HIVSmart! app and the Aspect^TM^ app; both mobile apps guide self-testers through the testing and reporting process. These apps were both tested in a clinical setting, under the observation of healthcare workers (HCWs), and whilst both the apps reported high usability and acceptability, they did not investigate the reporting of results in a non-clinical setting as an outcome.^16,17,18,19^

The Ithaka app (Aviro Health, Cape Town, South Africa) has been developed to close this gap by providing untrained HIVST users a mobile platform to self-report their HIVST results independent of a formal clinical setting, whilst also removing the potential for observational bias. The objective of this study was to investigate the use of Ithaka as an HIVST support tool for individuals, specifically the ability to report self-results outside a clinical environment.

## Methods

### Study design

This was a cross-sectional evaluation conducted from November 2018 to June 2019 with a random sample of 751 consenting adults from the general population of inner-city Johannesburg, South Africa. People who received an HIVST kit were invited to participate in the study. As per the HIVST programme, requirements to receive an HIVST kit were if they had willingness to perform an HIVST, had not tested for HIV in the previous 3 months, had a mobile phone compatible with the app, were 18 years or older, were able to read English and were able to provide written informed consent. Participants were excluded if they were known to be living with HIV, were a practising HCW or if they were taking drugs that could affect the sensitivity of the test, such as pre-exposure prophylaxis, ART or an experimental HIV vaccine. Before the study, a 2-week pilot period that included 41 people was used to improve operational issues, refine the content and user experience of the app and confirm the linkage between the data collection and data analysis datasets.

### App development

The Ithaka self-test support tool is a mobile phone-based tool to support users through self-testing and eventual confirmatory testing. It is a Progressive Web App (PWA), which is accessible as a reverse-billed mobi-site, where the provider pays any data costs, rendering the tool free to end users. The Ithaka platform provides users with a tailored journey to encourage user retention, reporting and linkage to care, as well as gamification to boost user engagement. Ithaka guides the patients through the various testing steps and will prompt the users to report back on their status, progress, emotional state, information comprehension and user satisfaction. Before conducting the self-test, users must complete a brief counselling component that explains the test process, and what to expect after obtaining the results; however, if users want more information, they can access integrated chat-based help at any time, or request a call back from a call centre. In the event of a positive HIV result, the study participant is referred for clinical treatment and care, whilst participants who test negative will be counselled and encouraged to seek confirmatory testing at 3 months.

The Ithaka platform is secure, with unique user profile logins and encrypted back-end databases to ensure data security and patient anonymity in line with the Protection of Personal Information (POPI) guidelines.^20^ Furthermore, stakeholders can receive real-time data on how users are engaging with the materials and platform. Screenshots of Ithaka are presented in Figure 1.

**FIGURE 1:**
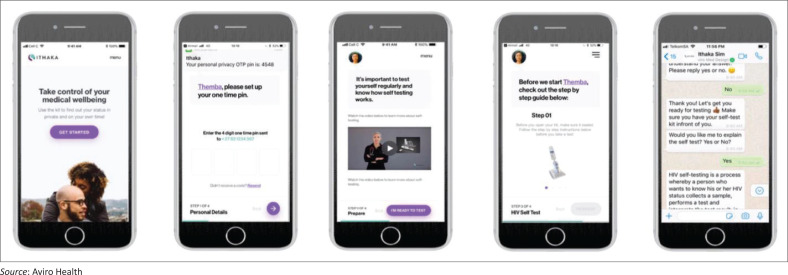
Ithaka screenshots.

### Data collection

HIV Self-Testing Africa (HSTAR) is an HIVST distribution and research programme that supplies free OraQuick® Rapid HIV Self-Test (Orasure Technologies, Bethlehem, USA) to people in Region F of Johannesburg, South Africa, through fixed-point distribution sites. These sites were also used to recruit participants for the Ithaka study. To collect a random sample and minimise disruption to the regular HSTAR programme (since recruitment was being performed by the distribution team), one random day each week was used to recruit participants for the Ithaka study. After an individual received their self-test, peer educators invited them to participate in the Ithaka study. No additional log was maintained to document individuals who declined to participate.

If an individual showed interest, the peer educator provided detailed information on the Ithaka study and obtained a written informed consent prior to administering the pre-survey questionnaire. The peer educator helped the participant log into and register on the app on the participant’s phone, which was available through a uniform resource locator (URL).

Data were collected from three sources as follows:

1. **Pre-study survey**: An in-person survey was conducted by peer educators to capture demographic information, including age, education, shared phone, gender and location.
2. **Ithaka platform**: The app tracked user engagement marked by logging on, registering, receiving counselling and reporting results.
3. **Post-study survey**: A telephone survey was conducted to obtain user feedback on the app, which included Likert-scale questions ([1] strongly disagree; [2] disagree; [3] neither agree nor disagree; [4] agree; [5] strongly agree) and open-ended questions. The Likert scale was used to understand the user experience of Ithaka (asking ratings on usefulness, ease of use, empowering, trustworthiness, ease of understanding and reliability), and whether it decreased barriers to report results, find a clinic, read frequently asked questions (FAQs), get reminders and make referrals. Participants were asked open-ended questions regarding their discontinued usage of the app, challenges using the app and if they would recommend it to a friend (Appendix 1). All participants were invited to participate in the post-study survey via a phone call to the number they had provided. Participants were eligible to participate if they provided consent and had completed the app journey, making it to the final reporting results stage, and answered all survey questions.

### Data analysis

Data from the surveys and Ithaka database were cleaned in Excel (Microsoft, Seattle, USA) and then exported to Stata V.14 (StataCorp, College Station, USA) for analysis. Demographic information and questions about app usage were described with frequency and percentages. User flow through the app was tracked and then presented with frequency and percentage through each stage. Likert scores were averaged and presented as a number between 1 and 5, with numbers approaching five representing favourable outcomes.

### Ethical consideration and approval

Ethics approval was obtained from the Human Research Ethics Committee of the University of the Witwatersrand, reference number: 180708. All participants provided written informed consent. The app was made available as a reverse-billed site, so participants did not incur data costs, but participants were provided no reimbursements for their time in the study.

## Results

### Demographics

A total of 751 people participated in the study. Nearly half of the participants, 340 (45.3%), were between the ages of 26 and 35 years, a third were 25 years old or below, 231 (30.8%) and about a quarter above were 35 years of age, 175 (23.3%). Four hundred and thirty-one (57.4%) participants were female, and 634 (84.4%) did not share mobile phones with anyone. Only 3 (0.4%) participants had a primary school education, 444 (59.1%) had a secondary school education and 203 (27.0%) had a tertiary school education, or higher. The complete demographic characteristics are presented in Table 1.

**TABLE 1:** Demographic characteristics.

Demographics	Frequency (*n* = 751)	Percentage† (%)
**Age (years)**		
18–25	231	30.8
26–35	339	45.1
Over 35	175	23.3
Not answered	6	0.8
**Sex**		
Female	431	57.4
Male	318	42.3
Not answered	2	0.3
**Highest level of education**		
None	4	0.5
Primary school education	3	0.4
Secondary school education	444	59.1
Tertiary school education	203	27.0
Not answered education	97	12.9
**Do you share a phone?**		
No	634	84.4
Yes	74	9.9
Not answered	43	5.7

*n*, number.

Note: †, The percentages may not add up to 100.0% because of rounding.

### Ithaka use

**FIGURE 2:**
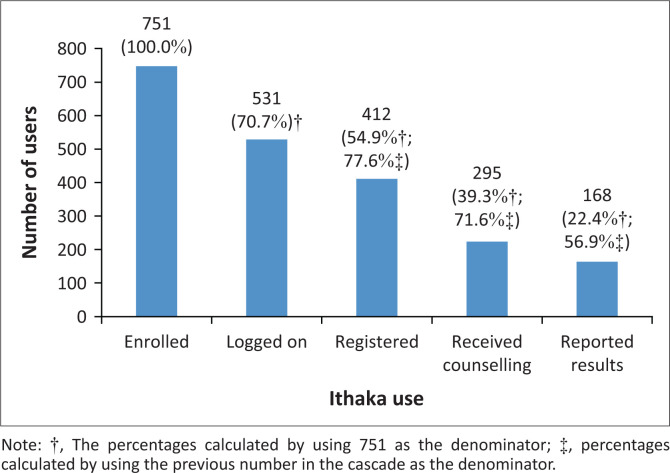
User journey through Ithaka: November 2018 to June 2019.

Figure 2 shows the cascade of Ithaka use from the point of enrolment to reporting HIV results. Approximately, three quarters, 531 (70.7%), logged on to the app. More than half the enrolled participants, 412 (54.9%), completed the registration process, 295 (39.3%) enrolled participants completed the pre-test counselling and the how-to-test instructions and 168 (22.4%) enrolled participants self-reported their results. Of the 168 participants who self-reported their results, 14 (8.3%) reported as HIV positive.

### Ithaka user experience

Of the 336 participants who were successfully contacted for the post-test telephone survey, consent to participate was provided by 190 (56.5%) participants, although only 112 (33.3%) were eligible for the post-study survey, and 41 (37.3%) completed the entire survey. To quantify the user experience, mean Likert scores approaching five represented strong agreement with the statement, whereas scores approaching one represented strong disagreement with the statement. The two statements, *Ithaka made it easy to upload results* and *Ithaka made it easy to find a clinic* had mean Likert scores of 3.8 (SD = 1.6) and 4.2 (SD = 0.9), respectively. All other user experience statements were strongly agreed with, receiving ratings that were above or equal to 4.5 (SD = 0.5–0.7). The mean Likert scores are presented with standard deviations (SDs) in Table 2.

**TABLE 2:** Mean Likert scores for user experience.

Outcome	Mean Likert score (*n* = 41)	SD
Overall, Ithaka was useful.	4.7	0.6
Overall, Ithaka was easy to use.	4.7	0.6
Overall, Ithaka made you feel enabled.	4.7	0.6
Overall, you trusted Ithaka.	4.7	0.5
Ithaka made it easy to upload results.	3.8	1.6
Ithaka made it easy to find a clinic.	4.2	0.9
The app language was easy to understand.	4.6	0.5
The information in the app was reliable.	4.6	0.7
The FAQs were helpful.	4.5	0.7
The reminder and referral functions were useful.	4.5	0.6

*n*, sample size; SD, standard deviation; FAQs, frequently asked questions.

When participants were asked why they had stopped using the app, 39/41 (95.1%) respondents stated that they used the app to completion, whilst two (4.9%) stated that they stopped because they were unable to upload their HIVST results. All 41/41 (100.0%) participants who responded stated that they would recommend the app to someone else, with respondents citing ease of use 12/41 (29.3%), liking the app 4/41 (9.8%) and privacy 2/41 (4.9%) as the main reasons for why they would recommend it to someone else. Most of the respondents, 36/41 (87.8%) stated that they did not experience any challenges or difficulties whilst using the app; however, 2/41 (4.9%) respondents cited data costs as a challenge, 2/41 (4.9%) respondents stated that they had difficulty uploading results and 1/41 (2.4%) respondent stated that he or she had experienced challenges because of the app languages. All user experience questions are presented in Table 3.

**TABLE 3:** Open-ended user experience questions.

Question	Frequency	Percentage†
**Why did you stop using the app? (*n* = 41)**		
Completed the survey at the end	39	95.1
Failed to upload results	2	4.9
**Would you recommend this app to someone else? (*n* = 41)**		
Yes	41	100.0
No	0	0.0
**Why would you recommend this app to someone else? (*n* = 41)**		
Easy to use	12	29.3
Liked the app	4	9.8
Privacy	2	4.9
Provides education	1	2.4
Language easy to understand	1	2.4
Reliable	1	2.4
Low data cost	1	2.4
No specific reason	19	46.3
**What was the biggest challenge whilst using the app? (*n* = 41)**		
No challenge	36	87.8
Language	1	2.4
Data costs	2	4.9
Uploading results	2	4.9

*n*, number.

Note: †, Percentages may not add up to 100.0% because of rounding.

## Discussion

To our knowledge, this is the first study in South Africa to evaluate the use of an mHealth app to self-report HIVST results as an outcome, independent of observation in a clinical setting. Previous feasibility studies have shown high acceptance of mHealth apps for the monitoring and evaluation of HIVSTs; however, they only evaluated usability in the presence of HCWs and did not evaluate any reporting outcomes through the app.^16,17,18,19^

Similar to these previous studies, the Ithaka app showed high self-reported usability amongst those interviewed, whilst also confirming that participants under real-world conditions were willing and able to self-report their results via the app. The self-reporting of results by logged on participants through the Ithaka app was 22.4%, which is acceptable, considering that it is common for apps to lose up to 80% of their active users in the first week.^21^

Furthermore, the percentage of HIVST results reported through Ithaka was more than twice that of a previous tele-health intervention in South Africa, which only led to 9.8% of participants self-reporting.^15^

Despite this increase in self-reporting and high usability Likert scores, 43.1% of participants who received counselling (a proxy for completing the self-test) still did not self-report their HIVST results, which leaves opportunity for improvement. Although field testing of Ithaka followed a 3-month human-centred design (including personal and journey mapping) and a 2-week pilot testing, a percentage (12.2%) of surveyed participants did experience challenges with the Ithaka platform. This not only suggests that users may need more than a brief introduction from a peer educator but also suggests that the technology development phase requires several iterations with greater consideration for pragmatic value propositions and testing of varied content or messaging before inclusion. Going forward, focus group or follow-up interviews with participants who did not complete the app journey could be conducted to further identify areas of improvement that caused participants to cease activity on the app.

Similar to reports of other South African digital health interventions, for users to completely embrace Ithaka and realise its full use, marketing campaigns can be used to create awareness, followed by a more comprehensive onboarding to motivate users.^22^ Although practical reasons for stopping the use of the app, such as forgetting to log back in or not using the test yet, should be mitigated with text message reminders, which have been shown to improve the user responsiveness of other mHealth apps,^23,24,25^ we did not find this in our study in which registered participants received reminder messages on day 1 and day 7. Some participants cited data costs and network issues as challenges to the app, and these are well-documented barriers for any mHealth app to enter into the South African market;^6,22^ however, Ithaka was a reverse-billed online platform that removed the barrier of data costs. As a reverse-billed platform, any and all data costs for using the platform are paid for by the service provider (Ithaka), and the end-users do not incur any costs, nor do they use any of their own data whilst on the platform.

There may have been some confusion by study participants regarding the meaning of reverse-billing, and this beneficial feature should be sufficiently explained to users in the future, so they know that no costs are incurred on their end whilst using the platform.

In South Africa, there is currently no endorsed platform for users to self-report their HIVST results, or be linked to care following a positive test,^6^ which makes the monitoring and evaluation very ineffective for the government and associated public health stakeholders.^7^ This study has shown that as a proof-of-concept, HIVST users are willing and able to self-report their HIVST results via the Ithaka app, and this sharing of information on a national scale could greatly improve HIVST monitoring and evaluation.

Whilst this study focussed on self-testing, which directly addresses the gap between the first 90 and the 85% of HIV-positive South Africans who know their status, it does not address the country’s largest deficit, as only 71% of people who are eligible for ART are actively receiving treatment.^26^ Ithaka could continue to increase active users by sending out reminders to encourage the self-reporting of results and keep users engaged by promoting linkage to care opportunities. To improve accessibility and usability, the Ithaka platform has since been extended to WhatsApp and to support blood-based tests. The Ithaka platform has also undergone a number of processes and content changes that were implemented as a way to continue improving on the HIVST reporting rate. In addition, extensions to the tool to support and confirm linkages to care and improve initiation and viral load suppression are currently undergoing piloting and development.

## Limitations

This study presented some limitations. Participants were recruited through existing HIVST distribution points, so individuals may have had previous exposure to HIVST studies, and potential study fatigue may have influenced their willingness to participate. Because of this exposure, participants may have a greater base-level background knowledge of HIVST than the general population. The Ithaka app was only available to individuals with mobile phones capable of running the current iteration of the app and does not include individuals who could not access the app because of different operating systems or memory capacity. Furthermore, a peer educator helped participants log into and register on the app, which may have influenced the ease of use and initial components of the cascade.

The use of only one HIVST kit means that these results also cannot be generalised across all HIVST kits.

Additionally, only 8.3% of participants self-reported an HIV-positive result, which is much lower than the national prevalence of 13.1%, and this may be because of a selection or reporting bias, where individuals who may be HIV positive did not participate or report their positive results. The views presented of the user experience responses may not represent the views of the study population as only participants who completed the app journey and answered all questions were included in the post-study survey results. The low completion rate for some of the survey questions represents a minority of the group and a larger minority in relation to the general population.

Lastly, the post-test survey was conducted via voice call, which may have attributed to this low completion rate.

## Conclusion

Millions of HIVST kits have been distributed globally; however, there is currently no universally accepted platform for users to self-report their HIVST results, health behaviour and outcomes in line with the HIV care cascade. This study has shown that HIVST users outside the clinical setting were willing and able to self-report their results via the app. This could be used on a national level to improve the monitoring and reporting of HIVST programmes, leading to the optimisation of kit distribution, and targeted marketing and support. The use of an app introduces the possibility to promote and improve linkage to care, counselling and follow-up for newly tested HIV-positive users. This, together with exploring other popular channels for making digital services available such as WhatsApp, needs to be explored further to ultimately enable the development of an app that is user friendly, cost efficient and beneficial to HIV programmes.

## Figures and Tables

**FIGURE 1-A1:**
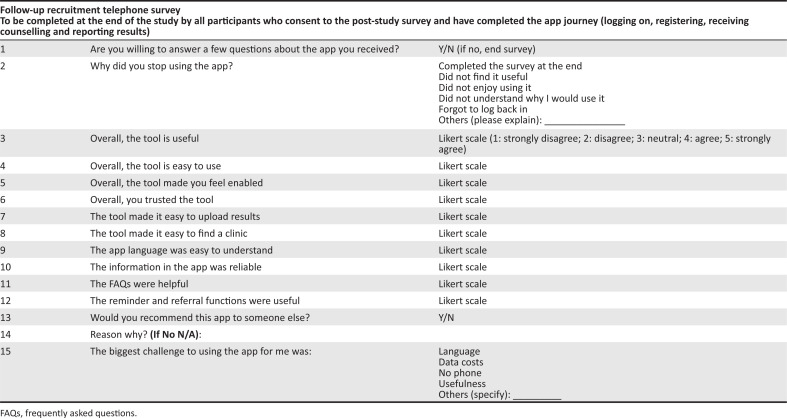
Post-study survey.
